# Value of implantable loop recorders in patients with structural or electrical heart disease

**DOI:** 10.1007/s10840-018-0354-y

**Published:** 2018-03-13

**Authors:** Rafi Sakhi, Dominic A. M. J. Theuns, Rohit E. Bhagwandien, Michelle Michels, Arend F. L. Schinkel, Tamas Szili-Torok, F. Zijlstra, Jolien W. Roos-Hesselink, Sing-Chien Yap

**Affiliations:** Department of Cardiology, Erasmus Medical Center, P.O. Box 2040, 3000 CA Rotterdam, The Netherlands

**Keywords:** Implantable loop recorder, Bradyarrhythmias, Ventricular arrhythmias, Risk stratification, Cardiomyopathy, Channelopathy, Congenital heart disease

## Abstract

**Purpose:**

In patients with structural heart disease (SHD) or inherited primary arrhythmia syndrome (IPAS), the occurrence of unexplained syncope or palpitations can be worrisome as they are at increased risk of sudden cardiac death. An implantable loop recorder (ILR) can be a useful diagnostic tool. Our purpose was to compare the diagnostic yield, arrhythmia mechanism, and management in patients with SHD, patients with IPAS, and those without heart disease.

**Methods:**

Retrospective single-center study in consecutive patients who underwent an ILR implantation.

**Results:**

Between March 2013 and December 2016, a total of 94 patients received an ILR (SHD, *n* = 20; IPAS, *n* = 14; no SHD/IPAS, *n* = 60). The type of symptoms at the time of implantation was similar between groups. During a median follow-up of 10 months, 45% had an ILR-guided diagnosis. Patients with IPAS had a lower diagnostic yield (14%) in comparison to the other groups (no SHD/IPAS 47%, *P* = 0.03; SHD 60%, *P* = 0.01, respectively). Furthermore, patients with SHD had a higher incidence of nonsustained VT in comparison to patients without SHD/IPAS (30 versus 3%, *P* < 0.01). ILR-guided therapy was comparable between groups. In the SHD group, a high proportion (10%) received an implantable cardioverter-defibrillator; however, this was not statistically significantly higher than the other groups (no SHD/IPAS 3%, IPAS 0%, *P* = 0.08).

**Conclusions:**

In comparison to patients without heart disease, the diagnostic yield of an ILR was lower in patients with IPAS and the prevalence of ILR-diagnosed nonsustained VT was higher in patients with SHD.

## Introduction

Implantable loop recorders (ILRs) are increasingly being used for the detection of infrequent arrhythmia episodes. Several studies have demonstrated the incremental value of ILRs over intermittent monitoring strategies for the detection of arrhythmias in patients with recurrent syncope, undocumented palpitations, and cryptogenic stroke [1–4]. ILRs might also be used as a diagnostic tool in patients at risk for ventricular tachyarrhythmias (VTs), such as those with structural heart disease (SHD) or inherited primary arrhythmia syndromes (IPAS) [5–7]. The occurrence of unexplained syncope or palpitations can be worrisome in these patients. The 2009 ESC syncope guidelines recommend considering an ILR in nonhigh-risk patients with SHD or IPAS [7]. The recent J-wave expert consensus report also suggests the use of an ILR for close monitoring of Brugada patients with presumed non-arrhythmogenic syncope [8]. There is limited data comparing the value of an ILR in patients with and without SHD [9, 10]. Considering the nature of the underlying disease, we hypothesized that patients with SHD/IPAS would have a higher incidence of ventricular arrhythmias than patients without an underlying heart disease. The purpose of the present study was to evaluate diagnostic yield, arrhythmia mechanism, and subsequent arrhythmia management in patients with and without SHD/IPAS receiving an ILR.

## Methods

### Study population

This observational cohort study involved consecutive patients who received an insertable ILR (Reveal LINQ, Medtronic Inc., Minneapolis, MN, USA) between March 2013 and December 2016 at our institution. The indication for the ILR was established by the treating physician and all patients gave informed consent for the implantation procedure. There were no patients who received an ILR for cryptogenic stroke. Patients with SHD included those with manifest heart disease at potential risk of arrhythmias including patients with coronary artery disease, inherited cardiomyopathy, infiltrative cardiomyopathy, and congenital heart disease. Patients with IPAS included those with long QT syndrome, Brugada syndrome, and catecholaminergic polymorphic VT. Carriers of a pathogenic mutation associated with cardiomyopathy or IPAS were also considered part of either the SHD or the IPAS group.

### ILR implantation and follow-up

ILR implantation was performed as recommended by the manufacturer using the incision and insertion tool. The device was implanted subcutaneously over the fourth intercostal space on the left hemithorax, either 45° or parallel relative to the sternal border. The incision was usually closed with one braided absorbable suture. After implantation, the patient received the remote monitoring device, as well as instructions about its use for nightly automated transmissions. Patients were discharged on the same day of implantation. Programming was optimized to maintain a high specificity, at the cost of sensitivity: detection of bradycardia (30 beats per minute; 8 beats), pause (4.5 s), and tachycardia (176 beats per minute; 16 beats). Atrial fibrillation (AF) detection was set to “AF only.” All devices were linked to the CareLink network for remote monitoring and all episodes (automatically recorded or patient-activated episodes) were transmitted on a daily basis.

Ten days after implantation, the patients were scheduled at the out-patient clinic to check their wound and interrogate their device. After this visit, patients were seen at the out-patient clinic every 6 months or earlier when necessary based on the transmitted episodes. The diagnosis was called ILR-guided if a symptom-rhythm correlation was established and/or if a VT was observed.

### Statistical analysis

Continuous data are presented as mean ± standard deviation if the data were normally distributed, or as median with interquartile range (25th and 75th percentile) otherwise. Categorical variables are presented by frequencies and percentages. Differences of continuous variables between groups were analyzed with the unpaired Student’s *t* test or the Kruskal-Wallis test, as appropriate. Differences between categorical variables were evaluated using the chi-square test. In the case of a statistical difference between groups, post hoc pairwise analysis was performed. Event rates were estimated with the Kaplan Meier method, and differences between event rates were compared by log-rank test. Paired comparisons were made using Cox regression analysis and described with hazard ratios and 95% confidence intervals. A *P* value < 0.05 was considered statistically significant. Statistical analyses were performed using SPSS version 21.

## Results

**Fig. 1:**
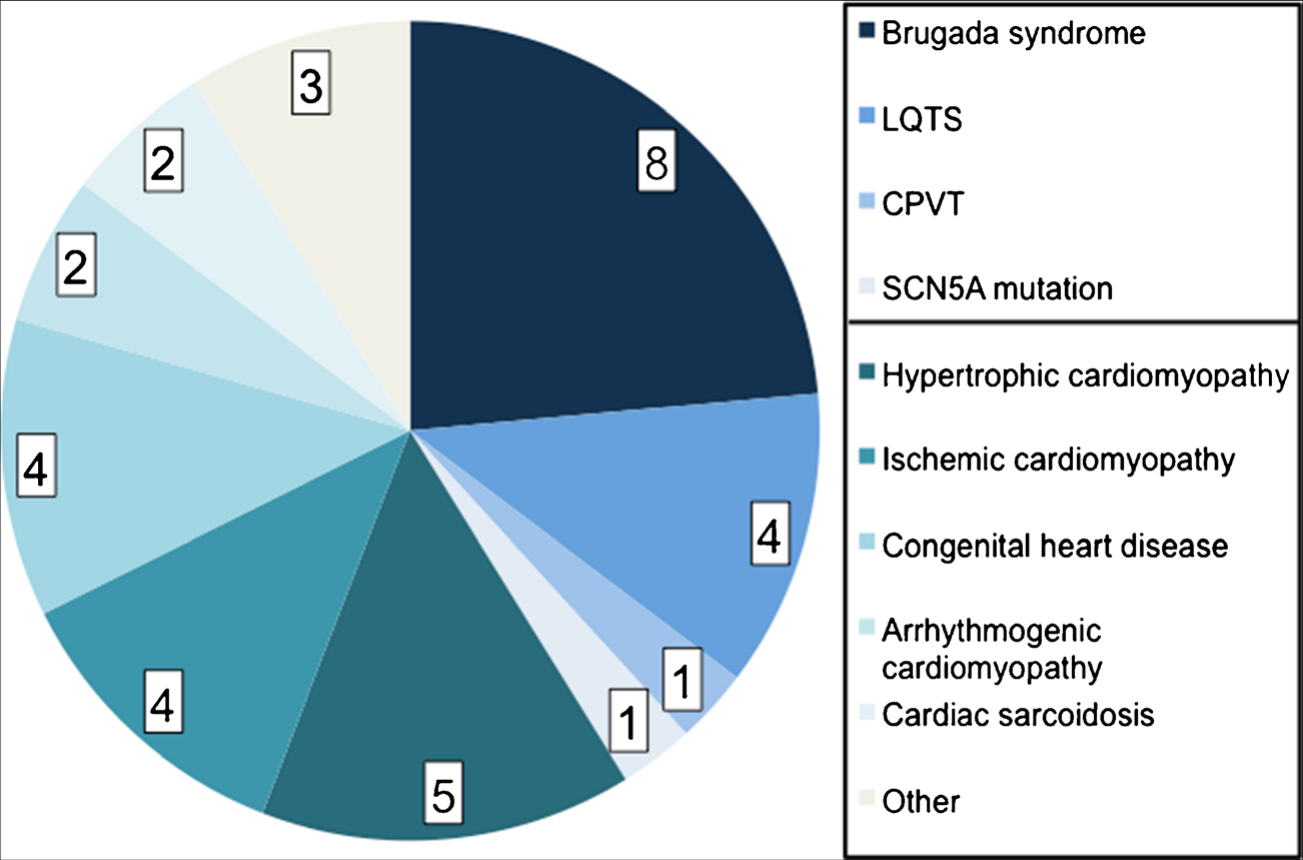
Overview of patients with structural heart disease or inherited primary arrhythmia syndrome. LQTS long QT syndrome, CPVT catecholaminergic polymorphic ventricular tachycardia

Between March 2013 and December 2016, a total of 94 patients underwent an ILR implantation. There were 20 patients (21%) with SHD and 14 patients (15%) with IPAS. Figure 1 provides an overview of the different underlying diagnoses in patients with SHD/IPAS. Except a higher proportion of PCI in the SHD group, there were no differences in baseline characteristics between groups (Table 1). It is also important to note that the presenting symptoms were similar between groups.

**Table 1:** Baseline characteristics

Variable	No SHD/IPAS *N* = 60	SHD *N* = 20	IPAS *N* = 14	*P* value
Demographics				
Age (years), mean ± SD	44 ± 17	47 ± 21	47 ± 11	0.73
Female gender, *n* (%)	36 (60)	10 (50)	8 (57)	0.74
Symptoms				
(Near) syncope, *n* (%)	47 (78)	14 (70)	10 (71)	0.71
Palpitations, *n* (%)	40 (67)	10 (50)	6 (43)	0.17
Asymptomatic, *n* (%)	–	1 (5)	1 (7)	0.15
Co-morbidity				
Hypertension, *n* (%)	6 (10)	4 (20)	3 (21)	0.37
Hypercholesterolemia, *n* (%)	8 (13)	2 (10)	1 (7)	0.79
Diabetes mellitus, *n* (%)	5 (8)	1 (5)	–	0.51
Transient ischemic attack, *n* (%)	4 (7)	1 (5)	–	0.61
Stroke, *n* (%)	2 (3)	2 (10)	–	0.11
Epilepsy, *n* (%)	2 (3)	–	2 (14)	0.11
Renal disease, *n* (%)	–	1 (5)	–	0.16
Prior PCI, *n* (%)	–	3 (15)	–	< 0.01
Prior CABG, *n* (%)	–	1 (5)	–	0.16

*CABG*, coronary artery bypass graft; *PCI*, percutaneous coronary intervention

**Fig. 2:**
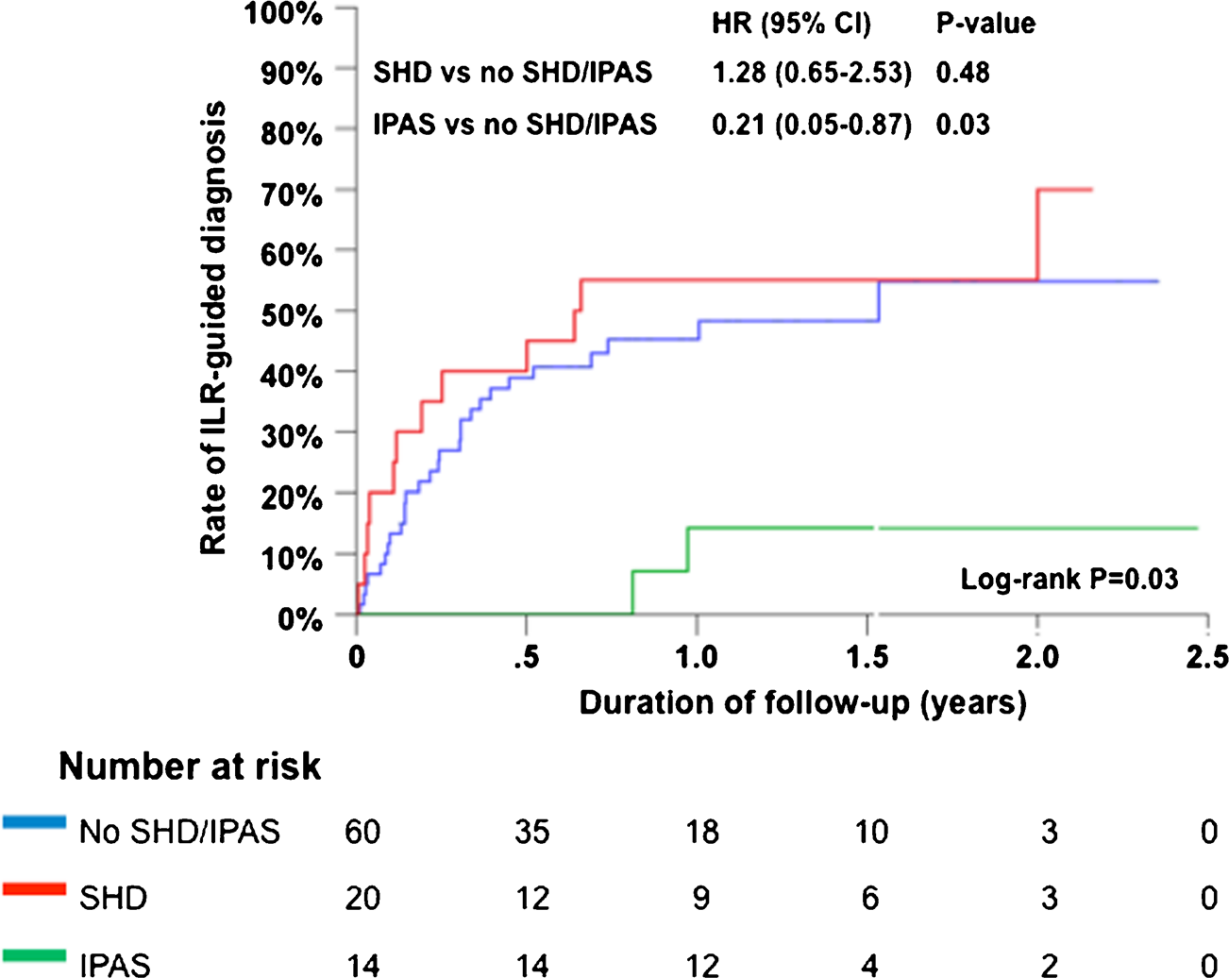
Cumulative event rate for ILR-guided diagnosis

During a median follow-up of 10 months (interquartile range, 3–17 months), 42 patients (45%) had an ILR-guided diagnosis. The diagnostic yield was different between groups (Fig. 2, Table 2). When performing pairwise comparisons, patients with IPAS had a lower diagnostic yield in comparison to patients with SHD (*P* = 0.01) or patients without SHD/IPAS (*P* = 0.03). Although patients with SHD and patients without SHD/IPAS had a similar diagnostic yield, the arrhythmia mechanism was different. Using pairwise comparison, patients with SHD had a higher incidence of nonsustained VT in comparison to patients without SHD/IPAS (*P* < 0.01).

**Table 2:** ILR-guided arrhythmia diagnosis

Diagnosis	No SHD/IPAS *N* = 60	SHD *N* = 20	IPAS *N* = 14	*P* value
Any arrhythmia diagnosis, *n* (%)	28 (47)	12 (60)	2 (14)	0.03
Sinus arrest, *n* (%)	6 (10)	1 (5)	1 (7)	0.77
Paroxysmal AV block, *n* (%)	1 (2)	1 (5)	–	0.56
Sinus bradycardia*, *n* (%)	2 (3)	–	–	0.56
Progressive ST, *n* (%)	2 (3)	–	–	0.56
Atrial fibrillation, *n* (%)	4 (7)	–	–	0.31
SVT, *n* (%)	9 (15)	2 (10)	–	0.28
Nonsustained VT, *n* (%)	2 (3)	6 (30)	1 (7)	< 0.01
Sustained VT, *n* (%)	2 (3)	2 (10)	–	0.31
No arrhythmia diagnosis, *n* (%)	32 (53)	8 (40)	12 (86)	0.03

*AV*, atrioventricular; *ST*, sinus tachycardia; *SVT*, supraventricular tachycardia; *VT*, ventricular tachycardia

*< 40 bpm for more than 10 s

Although there was a difference in the ILR-documented arrhythmia mechanism, the ILR-based therapy was similar between groups (Table 3). Most patients received antiarrhythmic drug therapy or had their antiarrhythmic drug dose increased. A high proportion (10%) of patients in the SHD group received an ICD; however, this was not statistically significantly higher than the other groups.

**Table 3:** ILR-based therapy

Therapy	No SHD/IPAS *N* = 60	SHD *N* = 20	IPAS *N* = 14	*P* value
Antiarrhythmic drug therapy, *n* (%)	9 (15)	7 (35)	2 (14)	0.14
Pacemaker, *n* (%)	9 (15)	3 (15)	–	0.31
Catheter ablation, *n* (%)	8 (13)	1 (5)	–	0.24
ICD, *n* (%)	2 (3)	2 (10)	–	0.08

*ICD*, implantable cardioverter-defibrillator

Four patients received an ICD; two of them had SHD. One patient with recurrent syncope and coronary artery disease with preserved ejection fraction received an ICD for sustained monomorphic fast VT. Another patient with hypertrophic obstructive cardiomyopathy received an ICD after experiencing nonsustained VT, which increased his estimated 5-year risk of SCD from 3.6 to 8.0%. Furthermore, two patients without SHD/IPAS received an ICD. One woman received an ICD for syncope and sustained monomorphic fast VT and another woman received an ICD after syncope and nonsustained polymorphic fast VT. No patient died suddenly during the study period.

## Discussion

The present study demonstrates that ILR patients with SHD have a higher incidence of nonsustained ventricular arrhythmias. However, there was only a trend towards a higher proportion of patients receiving an ICD in the SHD group in comparison to the other groups.

Studies which evaluated the performance of the ILR demonstrated a wide diagnostic yield ranging from 22 to 73% depending on the primary indication of the ILR [1, 3]. The diagnostic yield seems lower in patients with recurrent unexplained syncope than that in patients with undocumented palpitations. Overall, the diagnostic yield in our study population was 45%; however, patients with IPAS had a lower diagnostic yield. The diagnostic yield in patients with and without SHD (60 versus 47%) was similar in our study. A previous Austrian prospective ILR study in 70 patients with unexplained syncope (including 33 patients with SHD) found a similar diagnostic yield between patients with and without SHD (45 and 51%, respectively) [9].

Patients with SHD in our study experienced a high incidence of nonsustained ventricular arrhythmias, which is not surprising considering their predisposition to ventricular arrhythmias. A previous Italian ILR study in 103 patients with unexplained syncope (including 38 patients with SHD) also found a difference in arrhythmia mechanism between patients with and without SHD [10]. Patients with SHD were more likely to have paroxysmal/persistent AV block and tachyarrhythmias in comparison to patients without SHD. The incidence of ventricular arrhythmias was 5% in patients with SHD and 0% in patients without SHD [10]. Surprisingly, the incidence of ventricular arrhythmias in our IPAS group was low. This may be related to a lower threshold to implant an ILR in IPAS patients.

Although recurrent unexplained syncope is an established indication for an ILR, a rather novel indication is the use of an ILR for risk stratification [6, 7]. An EHRA survey demonstrated that 19% of centers use ILRs in patients with borderline indications for ICD therapy [5]. Currently, the ILR does not play a major role in the current guidelines on the prevention of SCD [11]. In the most recent guidelines, ILRs are recommended after comprehensive diagnostic evaluation when symptoms (e.g., syncope) are sporadic and suspected to be related to arrhythmias [11]. In some patients with SHD/IPAS, individual risk stratification can be difficult due to atypical symptoms. In these patients, long-term monitoring by an ILR may provide valuable information by the documentation of ventricular arrhythmias. Furthermore, it might also provide reassurance in these patients when they know that their symptoms are not related to ventricular arrhythmias. Considering the fact that a purely diagnostic tool is implanted in patients at risk for VTs, it is of importance to be alerted of a potential life-threatening episode as soon as possible. This is possible due to the availability of daily remote transmissions lowering the delay to medical intervention. No patient in our study died suddenly.

Several small ILR studies in patients with SHD or IPAS demonstrated that the proportion of ILR patients that received an ICD varies. Based on the available literature, no ILR patient with Brugada syndrome [12–14], long QT syndrome [15, 16], hypertrophic cardiomyopathy [9], or noncompaction cardiomyopathy [17] received an ICD during follow-up. In contrast, ICDs were implanted, based on the findings of the ILR, in patients with catecholaminergic polymorphic VT (11%) [15], congenital heart disease (0–9%) [16, 18, 19], Fabry cardiomyopathy (25%) [20], and SHD (0–28%) [9, 10, 21]. In our study, 10% of the SHD cohort received an ICD. Abovementioned data supports the use of ILRs in symptomatic patients with SHD for early detection of ventricular arrhythmias.

### Study limitations

The present study is small and the patient population is highly selected, thereby limiting the generalizability of the data. Furthermore, the lack of a control group (close follow-up with repeated ambulatory Holter monitoring) hampers conclusions on the incremental benefit of an ILR in comparison to alternative methods of monitoring. Therefore, all conclusions of the present study must be drawn with caution.
